# Editorial

**Published:** 1890-08

**Authors:** 


### Editorial.

### WILLIAM DAVID BIZZELL, M. D.

Dr. William David Bizzell died near Norcross, Ga., on June 30th, 1890, as he was beginning a journey through the mountains of Georgia and North Carolina in search of health. Though in a precarious state of health for some time past, the acute attack that ended his life was a great shock to his many friends. The immediate cause of his death was acute dysentery, though for some time he had been suffering from rapid tuberculosis of the lungs.

Dr. Bizzell was born in Alabama, May 31st, 1850, and after studying medicine under the pupilage of Dr. Williams, of Gainesville, Ala., entered the Medical College of Alabama, where he graduated with distinction in 1875, receiving the highest honor in his class. Locating in the city of Mobile, he became the Secretary of the Mobile Medical Society, and later one of the counsellors of the State Medical Association. He was subsequently elected Demonstrator of Anatomy, and later Professor of Chemistry in his *alma mater.* At this time he contributed many valuable papers to the Association, notably one on the "Climates of the United States Considered in Reference to the Treatment of Pneumonia and Consumption." In 1874 Dr. Bizzell was married to Miss Harriet Sims, of Mobile, who, with two daughters, survives him.

About eight years since he moved to Atlanta as the Medical Director of the Peoples' Mutual Insurance Company, and very soon he acquired a lucrative practice, which steadily increased until the time that he was compelled to leave the city on account of his failing health.

As a physician Dr. Bizzell deservedly occupied a high position in the estimate of the profession. Well educated, and a constant student, he commanded confidence as a consultant, and his counsels were often demanded. Soon after his residence in Atlanta, he was elected Professor of Chemistry in the Southern Medical College, and after serving in this chair for three years, he was promoted to the chair of Practice. In this he was a great success. A brilliant lecturer, as well as an efficient teacher, he was very popular with the classes, and his lectures were highly appreciated.

Personally he was very popular, and his affectionate and warm hearted nature drew to him many friends. These mourn his loss, and thus cut down in the beginning of his great usefulness, they can only think of the possibilities of his life had he been spared.

His colleagues in the faculty of the college recognize the loss that his death means to the institution, and his memory will long be cherished as one whose influence and labors meant much in the establishment of the school.

We can say nothing that will lessen the terrible burden resting upon his afflicted family. Leaving a record of which they may well be proud, his many friends join in deep and lasting sympathy for them in their affliction, and rejoice with them in the memory of what he was.

---

### TRIBUTE TO DR. BIZZELL.

At a meeting of the Atlanta Society of Medicine, held July 1st., 1890, the following resolutions were unanimously adopted:

WHEREAS, our esteemed and beloved fellow-member, Dr. W. D. Bizzell, has been removed from among us by the hand of death: therefore be it

Resolved, That in the death of Dr. Bizzell this Society has lost, not only an active and zealous member, and one who always strove, by word and deed, to promote its interests, but also a true-hearted and generous friend, who, in all the relations of life, fulfilled with earnestness of purpose and with kindness of heart every social obligation which was imposed upon him in his contact with his fellow men.

That this Society extends its heartfelt sympathy to his bereaved family, who have sustained in his death an irreparable loss.

That these resolutions be published in the daily papers and medical journals of this city, and that a copy be furnished to the family of the deceased.

### SOME NEW TERMS.

We are being constantly treated to new terms in current medical literature, and fortunate is the man who possesses a recent dictionary. In an article in the current issue of the New York *Medical Journal*, Dr. Robert T. Morris indulges in some departures in the way of description of the course of incisions. The following quotation will serve as a sample: "Hunter McGuire opens the bladder at the lowest available median ventrad point, and leaves the cephalad extremity of the abdominal incision open. The abdominal wound then being sutured elsewhere, we have a fistula left two or three inches in length, the walls of which are kept in apposition by the abdominal wall caudad to the external opening of the fistula, so that the patient can retain or pass his urine at will." We find again "dorsad." In looking over the dictionaries, the only one we have found containing these terms is the "Century," so we conclude that they have been recently introduced; certainly we have not before encountered them. A sensitive young woman who might accidentally see the description of a successful laparotomy that had been made upon her, might object to the dictionary telling her that the incision that opened the cavity was "a two-inch caudad incision," or, in other words, one two inches long, running "towards the tail." Yet such is the way that the new way would put it.

### A WISE SELECTION.

Dr. Willis F. Westmoreland has been unanimously elected to the chair of Surgery in the Atlanta Medical College.

This is a well-merited distinction worthily bestowed upon a rising young surgeon. We predict a brilliant career for the young professor. With the prestige of a noble ancestry, and an ambition to walk in the foremost rank, he will succeed. Thoroughly educated in his profession, cool, collected and deliberate in his undertakings, and possessed of all the elements of the true gentleman, he will prove an honor to the Atlanta Medical College. The appointment is not only a high compliment to the ability of Dr. W., but is a graceful tribute to the memory of his distinguished father, whom he succeeds.

### THE ENGLISH IDEA OF IT.

As a rule, the inhabitants of England are proverbially slow in taking hold of American ideas, but no stronger illustration of this is often found than appears in Keating's Cyclopedia of the Diseases of Children, just being published. In the article on Potts' Disease (page 1038, vol. III), the treatment by suspension and plaster jacket is accredited to American surgery, and the following "brief description" of the method of application is given: "Suspension is obtained by securing the head in a sling, which is attached to a strong cord playing in a pulley and fastened to a staple driven into a firm place above the patient's head. The patient having previously had a tight-fitting seamless shirt applied, the suspension is begun. The cord attached to the pulleys is so pulled that the patient's heels are raised from the floor." The application of the plaster then follows. It is to be hoped that no one will follow out these suspension rules, as the possibilities are too great to contemplate. Fortunately the author refers to the work of Dr. Sayre for a fuller description of the method. We can only wonder where the editor was when this went through the press.

### THE LEADER OF A REFORM.

The recently issued announcement of the Medical College of South Carolina inaugurates a change in the methods of Southern schools, in demanding in future a three years' graded course as a requisite to graduation. This change, instead of beginning with the session of 1892, as was agreed at the recent Nashville meeting, goes into effect at the opening of the coming term.

Too much praise cannot be accorded this old and efficient institution for this bold and aggresssive step, and we trust that the reward will be such as it deserves. It will, we believe, undoubtedly lessen the number of students at the coming session, but the Faculty will be repaid in the consciousness of having done the right thing, and having taken a step voluntarily, which must in the near future be forced upon all.

As a rule the average medical student of this section looks for the school where he can get his diploma cheapest, quickest, and with the least labor, so that colleges dependent for support upon the fees received for tuition have been compelled in self-defense to demand a co-operation of schools in such a radical reform, and this fact entitles the Charleston school to much credit for taking the initiatory. We only trust that the good example will be followed by others, and in the meantime we extend to the Medical College of the State of South Carolina our most distinguished consideration.

---

### THE WOMAN'S MEDICAL COLLEGE OF GEORGIA.

This institution begins its second annual term October 1st next, under the Presidency of Dr. A. G. Thomas, of this city. The class will consist exclusively of ladies. Half rates to wives and daughters of physicians, clergymen and Confederate veterans. For information, address J. W. Stone, M. D., box 215, Atlanta, Ga.

---

Just as we go to press we learn, with deep regret, of the death of Dr. R. C. Word, one of the former editors of this journal. A more extended notice will appear in our next.

---

The great question now is, "should clergymen use tobacco?" We think not. The clergy is overworked testing and testimonialising patent medicines. We shouldn't expect too much even of the clergy.—*Medical Advance.*

